# TGF-β1 reduces the oxidative stress-induced autophagy and apoptosis in rat annulus fibrosus cells through the ERK signaling pathway

**DOI:** 10.1186/s13018-019-1260-4

**Published:** 2019-07-29

**Authors:** Binbin Ni, Hao Shen, Wei Wang, Hua Lu, Leisheng Jiang

**Affiliations:** Department of Orthopaedic Surgery, Xinhua Hospital Affiliated to Shanghai Jiaotong University School of Medicine, No. 1665 Kongjiang Road, Shanghai, 200092 China

**Keywords:** Hydrogen peroxide, Autophagy, Apoptosis, ERK, Annulus fibrosus cells, Glutathion peroxidase-1, Intervertebral disc degeneration

## Abstract

**Background:**

The aim of this study is to explore the effects of TGF-β1 on autophagy and apoptosis induced by exogenous hydrogen peroxide (H_2_O_2_) in annulus fibrosus (AF) cells and possible signal pathways involved in this process.

**Methods:**

AF cells were isolated from rat lumbar discs and subjected to different concentrations of exogenous H_2_O_2_ (50, 100, 200 μmol/L) for different time periods (0.5, 1, 2, and 4 h). Cell viability was determined by CCK-8 assay, and the levels of autophagy and apoptosis were evaluated by Western blotting and caspase 3, 8, 9 activity assay. By administration with different concentrations of TGF-β1 (5, 10, 20 ng/mL), the effects of TGF-β1 on autophagy and apoptosis induced by H_2_O_2_ were observed, and the possible signaling pathways were also investigated by using various apoptosis inhibitors or an autophagy inhibitor Bafilomycin A (Baf A) in AF cells.

**Results:**

H_2_O_2_ significantly impaired cell viability in a dose- and time-dependent manner. H_2_O_2_ also induced a sudden and the highest level of autophagy at 1 h, and gradually increased apoptosis through ERK pathway. The mitochondrial pathway was involved in H_2_O_2_-induced apoptosis in AF cells. TGF-β1 reduced the expression of p-ERK and downregulated the expressions of Beclin-1, LC3 II/I, and mitochondrial-related apoptotic proteins (Bax/Bcl-2, caspase-9). Meanwhile, TGF-β1 downregulated the level of intracellular H_2_O_2_ through upregulating the expression level of glutathione peroxidase-1 (GPx-1).

**Conclusions:**

TGF-β1 reduced autophagy and apoptosis induced by exogenous H_2_O_2_ through downregulating the expression of ERK in AF cells. TGF-β1 could downregulate the level of ERK and intracellular H_2_O_2_ by upregulating GPx-1.

## Background

Chronic lower back pain (LBP) is an extremely common musculoskeletal disorder that affects approximately 80% of the population during their life [1]. LBP is associated with significant disability, which, on the one hand, reduces the health-related quality of life of patients; and, on the other hand, negatively impacts productivity and imposes a substantial economic burden on society and family [1]. Thus, understanding the attributions of LBP has gain increasing attention recently.

Although the etiology of LBP remains unclear, intervertebral disc degeneration (IVDD) is suggested as a main contributor. Annulus fibrosus (AF) is an important component of the intervertebral discs. During the degeneration of the intervertebral discs, the AF has been proven to be gradually lost due to apoptosis that consequently leads to its inability for withstanding to loading and induces the annular tears, fissures, and subsequent disc protrusions/herniations, ultimately resulting in the development of LBP [2–5]. Thus, investigations of mechanisms of AF cell apoptosis may be of importance to develop effective strategies for IVDD and LBP.

Accumulating evidence has reported that there was excessive generation of reactive oxygen species (ROS), including superoxide anion, hydrogen peroxide (H_2_O_2_) and hydroxyl radicals, nitric oxide (NO), etc. in degenerated intervertebral discs [6, 7]. ROS may mediate the pro-apoptosis effects on AF cells by increasing mitochondrial damage with decreased mitochondrial membrane potential (MMP) [8, 9] and inducing the secretion of pro-inflammatory cytokines [7, 10] to enhance the autophagy [11, 12]. Hereby, inhibition of ROS-mediated apoptosis may be underlying approaches for treatment of IVDD and LBP.

Transforming growth factor-β1 (TGF-β1) is a well-known factor to promote the cell growth and thus may be antagonistic to apoptosis of AF cells and alleviate the development of IVDD. This hypothesis has been preliminarily demonstrated by previous studies. Matsunaga et al. found that the expression of TGF-β1 was gradually decreased in AF cells of aging-induced IVDD model and almost disappeared in the 50-week-old mice [13]. The addition of TGF-β1 could reduce apoptosis via inhibiting nutrition deprivation-induced autophagy in rat AF cells [14] and reverse degeneration of intervertebral discs [15]. However, whether TGF-β1 also can inhibit ROS-mediated autophagy and then apoptosis in AF cells has not been investigated. The goal of this study was to observe the roles of TGF-β1 on autophagy and apoptosis in rat AF cells which was treated with exogenous H_2_O_2_.

## Materials and methods

### Isolation and culture of AF cells

All experimental protocols were approved by the Animal Care and Use Committee of Shanghai Jiaotong University and were in accordance with the guidelines for the Care and Use of Laboratory Animals published by the US National Institutes of Health. After male, 6-week-old Sprague-Dawley rats were sacrificed by intraperitoneal administration of 10% chloral hydrate (3.5 mL/kg, Sigma, Missouri, USA), the AF tissues were immediately excised from L1 to L6 intervertebral discs under aseptic condition. Primary AF cell cultures were prepared as our previously described [16, 17]. Briefly, AF tissues harvested were cut into small pieces (< 1 mm^3^) and firstly digested with 0.4% pronase (Calbiochem, San Diego, CA) for 90 min at 37 °C, followed by second-digestion with 0.025% collagenase type II (Sigma) and 0.01% hyaluronidase type V (Sigma) overnight. The suspension was filtered through a 70-μm nylon mesh filter to remove the tissue debris. Then, AF cells were seeded into culture plates and maintained at 37 °C under a humidified atmosphere of 95% air and 5% CO_2_. The medium was changed every 2 days. First-passage cells in a monolayer were used throughout the experiments.

### Cell treatment

AF cells were seeded into 96-well culture plates at a density of 5 × 10^3^ cells and maintained in complete medium (DMEM/F12 with 10% fetal bovine serum; Gibco/Invitrogen, Carlsbad, CA, USA). When the AF cells reached to 90% confluence, the medium was replaced with complete medium consisting of different concentrations of H_2_O_2_ (50, 100, 200 μmol/L; 30% solution, Sangon Biotech, Shanghai, China) for various time points (0.5, 1, 2, and 4 h). Each different concentration was repeated in 5 wells. To observe the influence of TGF-β1 addition on AF cells, different concentrations of TGF-β1 (5, 10, 20 ng/mL; Peprotech, Rocky Hill, NJ, USA) were added when the above pre-designed concentrations of H_2_O_2_ were reached. The cells were pretreated by various chemical blockers, including EK1/2 inhibitor U0126 (Calbiochem, San Diego, CA, USA), p38 inhibitor SB203580 (Calbiochem, San Diego, CA, USA), JNK inhibitor SP600125 (Calbiochem, San Diego, CA, USA), autophagy inhibitor Bafilomycin A (Baf A; Biotime, Shanghai, China), apoptosis inhibitors Z-VAD-FMK (BioVision, California, USA), Z-ATAD-FMK (BioVision, California, USA), and NS3694 (Calbiochem, Billerica, MA, USA), at 2 h before the addition of H_2_O_2_ and/or TGF-β1.

### Cell viability assay

Cell viability was measured using a Cell Counting Kit (CCK-8; Dojindo, Kyushu, Japan). In brief, 10 μL of CCK-8 solution was added to each well and incubated for 2 h in a humidified CO_2_ incubator at 37 °C. Automicroplate reader (Bio-Rad, Hercules, CA, USA) was used to measure the absorbance at a wavelength of 450 nm. Cell viability was expressed as the percentage of viable cells relative to untreated cells.

### Western blotting analysis

When 90% confluence was reached, the AF cells were lysed in radio-immunoprecipitation assay buffer. The protein concentration of the whole cell lysate was measured by bicinchoninic acid protein assay kit (Pierce, Rockford, IL, USA). A total of 40 μg total protein from different samples was electrophoresed in different concentrations (6%, 10%, 12%) of sodium dodecyl sulfate polyacrylamide gel and electro-transferred to a polyvinylidene difluoride membrane (Beyotime, Shanghai, China). After being washed for three times with 10 mM Tris-buffered saline with 1.0% Tween-20, the polyvinylidene difluoride membranes were incubated with 5% dehydrated skim milk to block nonspecific protein binding and then incubated with primary antibodies against PARP (Cell Signaling Technologies, Beverly, MA, USA; #9548, 1:1000 dilution), cleaved caspase-3 (Cell Signaling Technologies, Beverly, MA, USA; #9964, 1:1000 dilution), Bax (Cell Signaling Technologies, Beverly, MA, USA; #2772, 1:1000 dilution), Bcl-2 (Cell Signaling Technologies, Beverly, MA, USA; #2870, 1:2000 dilution), Beclin-1 (Cell Signaling Technologies, Beverly, MA, USA; #3495, 1:1000 dilution), LC3 II/I (Cell Signaling Technologies, Beverly, MA, USA; #3868, 1:500 dilution), SQSTM1/p62 (Cell Signaling Technologies, Beverly, MA, USA; #5114, 1:1000 dilution), T-P44/42 MAPK-ERK1/2 (Cell Signaling Technologies, Beverly, MA, USA; #4695, 1:1000 dilution), SAPK/JNK (Cell Signaling Technologies, Beverly, MA, USA; #9258, 1:1000 dilution), p38 (Cell Signaling Technologies, Beverly, MA, USA; #8690, 1:1000 dilution), phospho-P44/42 MAPK-ERK1/2 (Cell Signaling Technologies, Beverly, MA, USA; #4370, 1:1000 dilution), phospho-SAPK/JNK (Cell Signaling Technologies, Beverly, MA, USA; #4671, 1:1000 dilution), phospho-P38 (Cell Signaling Technologies, Beverly, MA, USA; #9215, 1:1000 dilution), cytochome C (Proteintech, Chicago, USA; 10993-1-AP, 1:1000 dilution), caspase-9 (Proteintech, Chicago, USA; 10380-1-AP, 1:1000 dilution), caspase-12 (R&D, Minneapolis, Minnesota, USA; AF1456, 0.5 μg/mL dilution), GPx-1 (R&D, Minneapolis, Minnesota, USA; AF3798, 1 μg/mL dilution), and β-actin (Cell Signaling Technologies, Beverly, MA, USA; #8457, 1:500 dilution) in the diluent (Beyotime, Shanghai, China; #P0023A) at 4 °C overnight, respectively. Subsequently, the membranes were incubated with horseradish peroxidase-conjugated goat anti-rabbit IgG secondary antibodies (Dako, Carpinteria, CA, USA; 1:1000~1:2000 dilution). Protein bands were visualized by the Super Signal Chemiluminescent Substrate system (Millipore or BeyoECL Plus) and quantified by densitometry analysis using the Bio-Rad Image Lab 2.0 software (Bio-Rad Laboratory, Hercules, CA, USA).

### Caspase-3, caspase-8, and caspase-9 activity detection

The activities of caspase-3, -8, and -9 were detected by using colorimetric activity assay kits (Beyotime, Shanghai, China; #C1116, #C1152, #C1158), respectively. Cells were incubated in a lysis buffer containing 1% Dithiothreitol (DTT) on ice for 15 min and then centrifuged at 20,000 rpm for 15 min to isolate proteins. The protein concentration was determined using Bradford protein assay. A total of 200 μg protein lysate was incubated with 5 μL of caspase-3, -8, or -9 substrate and 1% DTT in the dark for 4 h at 37 °C. The activities of caspase-3, -8, and -9 were evaluated using a spectrophotometer at 405 nm with a microplate spectrophotometer (Biotek Instruments, Winooski, VT, USA). According to their concentration curve, the value was expressed by A_405_ (measured in each process hole)/A_405_ (measured in control hole).

### Mitochondrial membrane potential measurement

The AF cells harvested were re-suspended in a mixture of 500 μL culture medium and 500 μL fluorescent dye 5,5′6,6′-tetrachloro-1,1′,3,3′-tetraethylbenzimidazol-carbocyanine iodide (JC-1) (Beyotime, Shanghai, China; #C2006), and then incubated in the dark at 37 °C for 20 min. Subsequently, the re-suspended cells were subjected to flow cytometry to determine the MMP (mtΔψ) which was measured by the dual-emission potential-sensitive probe. Cells with normal mitochondria had a high mtΔψ, JC-1 formed orange-red fluorescent J-aggregates; while in cells with depolarized or damaged mitochondria, the sensor dye appeared as green fluorescent monomers. The value of mtΔψ from each sample was expressed as the ratio of red fluorescence intensity/green fluorescence intensity.

### Apoptosis detection

Annexin V-FITC/PI apoptosis detection kit (BD Pharmingen, San Diego, CA, USA) was used to detect the apoptotic cells. The cells were collected by centrifugation (5 min, 1000 rpm) and re-suspended in 1× binding buffer at a concentration of 1 × 10^6^ cells/mL. Following that, 100 μL of the solution (1 × 10^5^ cells) was transferred to a 5 mL tube which was also added with 5 μL of Annexin V-FITC and 5 μL of PI. The cells were gently vortexed and incubated for 15 min at room temperature in the dark. Then, another 400 μL of 1× binding buffer was added into each tube, and flow cytometry analysis was performed within 30 min. The apoptosis incidence was calculated by the percentage of early apoptotic (Annexin V+/PI) cells plus the percentage of late apoptotic (Annexin V+/PI+) cells.

### Autophagy level detection

AF cells were incubated at a density of 2 × 10^5^ on 24-well plates under complete medium cultivation. After 60–70% confluence was reached, cells were transfected with 2 mg/mL GFP-LC3 (kindly provided by Dr. Li Wang, Neonatology, Shanghai Jiaotong University) or GFP-vector plasmid DNA using Lipofectamine 2000 (Invitrogen, Carlsbad, CA, USA). After incubation in Opti-MEM medium for 6 h, the cells were incubated in complete culture medium again for 24 h. The cells were fixed with 4% paraformaldehyde, and washed twice in cold PBS. Autophagy was evaluated by analyzing the formation of green fluorescent puncta of autophagosomes in GFP-LC3 transfected cells under the fluorescent microscope.

### Measurement of H_2_O_2_ content, catalase, and glutathione peroxidase activities

The total proteins or supernatants extracted from different examples were subjected to assay of catalase (Beyotime, Shanghai, China, #S0051) and glutathione peroxidase (GPx-1) (Beyotime, Shanghai, China, #S0056) activities as well as intracellular levels of H_2_O_2_ (Beyotime, Shanghai, China, #S0038) according to the manufacturer’s instructions.

### Statistical analysis

All data were expressed as mean ± standard deviation after at least three separate experiments and analyzed with SPSS13 Statistical Software (SPSS Inc., IL, USA). Differences between groups were analyzed using one-way ANOVA and independent Student’s *t* test in variables normally distributed, while Kruskal-Wallis and Mann-Whitney *U* test were adopted for variables with non-normal distribution. A *P* value < 0.05 was considered to be statistically significant.

## Results

### TGF-β1 reduces the H_2_O_2_-induced cytotoxicity

**Fig. 1:**
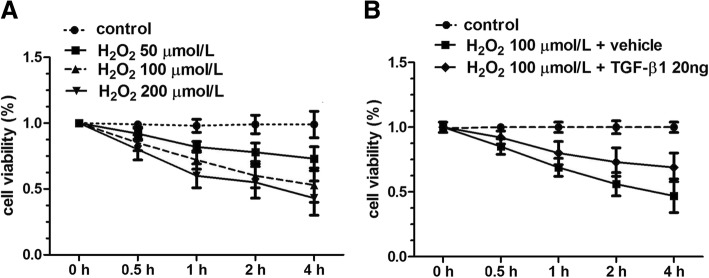
TGF-β1 reduces the H_2_O_2_-induced cytotoxicity. **a** Cell viability of annulus fibrosus cells after treatment with the increasing dosage of the H_2_O_2_ (50, 100, 200 μmol/L) and incubation time (0, 0.5, 1, 2, and 4 h). **b** Cell viability of AF cells after treatment with 100 μmol/L H_2_O_2_ and then incubation with 20 ng TGF-β1 for 0, 0.5, 1, 2, and 4 h. *TGF*-*β1* transforming growth factor-β1, *H*_2_*O*_2_ hydrogen peroxide

After being treated with different concentrations of H_2_O_2_ at different time points, the activity of AF cells was found to be significantly decreased, indicating its cytotoxicity. More importantly, the cell viability of AF cells were shown to be gradually declined with the increasing dosage of the H_2_O_2_ (50, 100, 200 μmol/L) and the prolongation of stimulation time (0.5, 1, 2, 4 h). After incubation with 50 μmol/L H_2_O_2_ for 4 h_,_ the cells that survived still could reach 79%; but nearly half of the AF cells died using 100 μmol/L H_2_O_2_ (55%); toxic effect of H_2_O_2_ on AF cells was more obvious following treatment with 200 μmol/L H_2_O_2_ (43%). These findings indicated the toxic effects of H_2_O_2_ exhibited time- and dose-dependent patterns (Fig. 1a).

In order to verify whether TGF-β1 could reduce the cytotoxicity induced by H_2_O_2_, TGF-β1 was added to AF cells after incubation with 100 μmol/L H_2_O_2_. As expected, different concentrations of TGF-β1 (5, 10, and 20 ng/mL) were demonstrated to partially reverse the cytotoxic effects of H_2_O_2_ and improve the cell viability of AF cells to 59%, 67%, 76% at 4 h, respectively (Fig. 1b). The results implied that the protective effects of TGF-β1 on H_2_O_2_-induced cytotoxicity were in a dose-dependent manner.

### TGF-β1 attenuates the H_2_O_2_-induced autophagy in AF cells

**Fig. 2:**
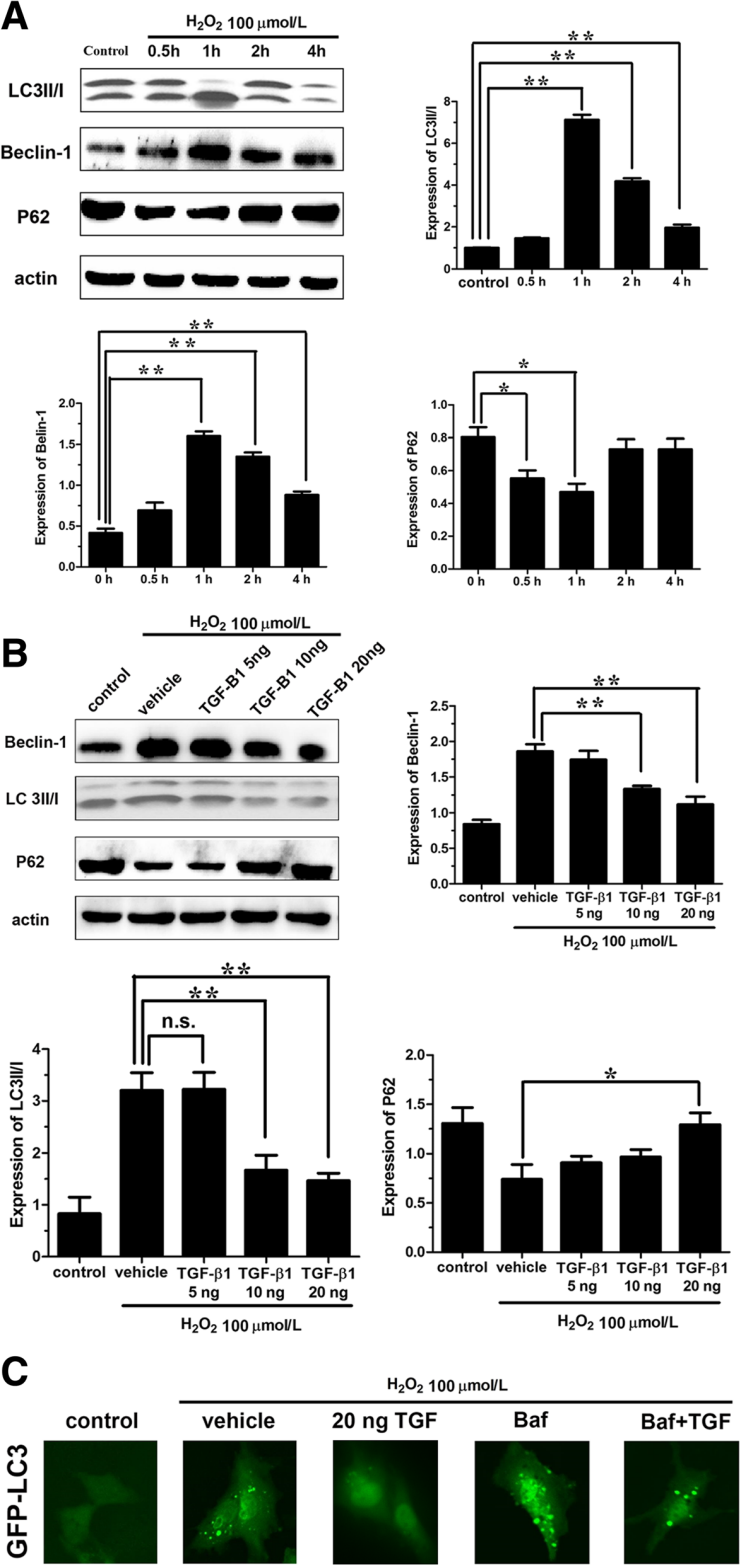
TGF-β1 attenuates the H_2_O_2_-induced autophagy in annulus fibrosus cells. **a** Western blot analysis to indicate the expression of autophagy-related proteins (Beclin-1 LC3 II/I, and p62) in annulus fibrosus cells after treatment with 100 μmol/L H_2_O_2_. **b** Western blot analysis to indicate the expression of autophagy-related proteins (Beclin-1, LC3 II/I, and p62) in annulus fibrosus cells after treatment with 100 μmol/L H_2_O_2_ and TGF-β1. **c** Green fluorescence to observe the GFP-LC3 in autophagosomes after treatment with TGF-β1 and autophagy inhibitor Baf. TGF-β1, transforming growth factor-β1. *H*_2_*O*_2_ hydrogen peroxide, *Baf* Bafilomycin, *n*.*s* not significant; *P < 0.05; **P < 0.001

To investigate the underlying mechanisms of H_2_O_2_ treatment on cell viability in AF cells, autophagy was analyzed. The results showed that after stimulation with 100 μmol/L H_2_O_2_ for 0.5 h, the levels of autophagy-related proteins Beclin-1 and LC3 II/I began to increase in AF cells, and reached their peak after 1 h (*P* < 0.01), thereafter gradually declined at 2 and 4 h (Fig. 2a). The expression of p62, which can be degraded by autophagic process, had a negative correlation with autophagy. Thus, the level of p62 began to reduce after stimulation by H_2_O_2_ for 0.5 h in AF cells (*P* < 0.05). When the autophagic level reached peak (expressions of Beclin-1 and LC3II/I were highest), the level of p62 trimmed down to the minimum (*P* < 0.05); thereafter, p62 gradually increased with the decrease of autophagy (Fig. 2a). These results confirmed that autophagy could be rapidly induced by H_2_O_2_ within 1 h, and then gradually reduced.

To study the roles of TGF-β1 on autophagy induced by H_2_O_2_, different concentrations of TGF-β1 (5, 10, 20 ng/mL) and 100 μmol/L H_2_O_2_ were used to incubate with the AF cells for 1 h. Western blot analysis showed that 10 and 20 ng/mL TGF-β1 could reduce the expressions of autophagy-related protein Beclin-1 and LC3 II/I, which was induced by H_2_O_2_, by a dose-dependent manner (*P* < 0.01) (Fig. 2b). Although the expression level of p62 gradually increased with the increasing concentration of TGF-β1, only statistical significance was observed when the cells were treated by 20 ng/mL TGF-β1 (*P* < 0.05) (Fig. 2b). The results demonstrated that high dose TGF-β1 may inhibit autophagy induced by H_2_O_2_, through decreasing the degradation of p62 and LC3 II/I transformation. In addition, GFP-LC3 was used to further validate the effects of TGF-β1 on autophagy induced by H_2_O_2_. Enhanced autophagy could be detected by fluorescence microscopy for increasing uptake of GFP-LC3 in autophagosomes which showed strengthened intensity of green fluorescence. The results showed that the AF cells were successfully transfected by GFP-LC3 and the fluorescence intensity was low in control group. After stimulation with 100 μmol/L of H_2_O_2_ for 1 h, the fluorescence intensity of AF cells was increased and could be suppressed by 20 ng/mL TGF-β1 (Fig. 2c). Baf A is an inhibitor of autophagy that can inhibit the degradation of autophagic contents (including GFP-LC3) in autophagosomes through blocking the combination of autophagosomes and lysosomes. For this reason, when autophagy was inhibited by Baf A, the decreased degradation of LC3 II resulted in its strengthened intensity of green fluorescence. Our results also found that Baf A enhanced GFP-LC3 fluorescence compared with the control group, and 20 ng/mL TGF-β1 could reduce the expression of GFP-LC3. Meanwhile, the level of GFP-LC3 elevated by Baf A could be suppressed by TGF-β1 which further demonstrated that autophagy was inhibited by TGF-β1 (Fig. 2c).

### TGF-β1 inhibits the H_2_O_2_-induced apoptosis in AF cells

**Fig. 3:**
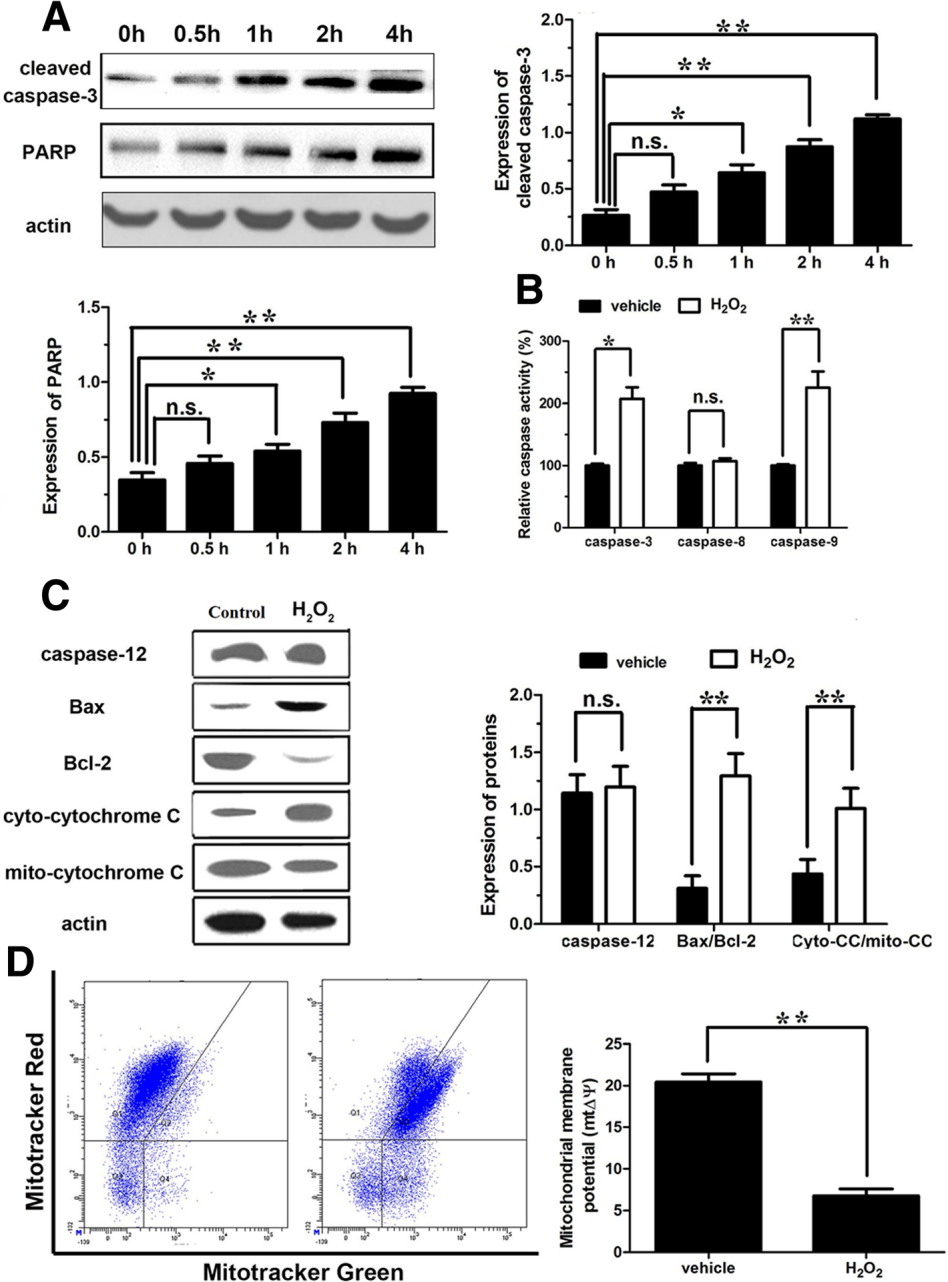
H_2_O_2_-induced apoptosis in annulus fibrosus cells. **a** Western blot analysis to indicate the expressions of cleaved caspase-3 and PARP. **b** The enzymatic activity test for caspase-9, caspase-3, and caspase-8. **c** Western blot to show the expression of caspase-12, Bax, Bcl-2, and cytoplasmic/mitochondrial cyt-C. **d** Mitochondrial membrane potential. *n*.*s* not significant; *P < 0.05; **P < 0.001

Another important form of programmed cell death, apoptosis was also analyzed to reveal the potential mechanisms of H_2_O_2_ treatment on cell viability in AF cells. Western blot was performed to detect the expressions of apoptotic markers cleaved caspase-3 and PARP. The results showed that the increase in the expressions of cleaved caspase-3 and PARP had been shown a statistical significance (*P* < 0.05) after AF cells were stimulated by 100 μmol/L H_2_O_2_ for 1 h and the highest level was achieved at 4 h (*P* < 0.01) (Fig. 3a), indicating that H_2_O_2_ could obviously induce the apoptosis of AF cells in a time-dependent manner.

As we know, there are many intracellular signaling pathways that can activate caspase-3 and excite downstream apoptotic processes in different cells: (1) oxidative stress could activate caspase-9, which belongs to mitochondrial pathway, and in turn activates the caspase-3 to execute the final apoptotic process [18]; (2) endoplasmic reticulum stress process mediated by caspase-12 could also activate caspase-3 [19]; and (3) caspase-8 activated by death receptor pathway could also activate caspase-3 to involve in the process of apoptosis [20]. In order to distinguish the downstream signaling pathways of H_2_O_2_-induced apoptosis in AF cells, enzymatic activity and apoptotic markers were further examined at 1 h after 100 μmol/L H_2_O_2_ stimulation. The test of enzymatic activity found that caspase-9 and caspase-3 activities were significantly increased (respectively, *P* < 0.01 and *P* < 0.05). Caspase-8 was slightly elevated (112%), but no statistical significance was observed (Fig. 3b). Western blot confirmed that the expression of caspase-12 was also slightly elevated, without statistical significance (Fig. 3c). These results showed that H_2_O_2_-induced apoptosis was mainly through the mitochondrial pathway. Meanwhile, Western blot analysis showed that H_2_O_2_ significantly increased the level of Bax/Bcl-2 (*P* < 0.01) (Fig. 3c), but dramatically decreased MMP (*P* < 0.01) (Fig. 3d) and released mitochondrial cyt-C to cytoplasm, which caused the value of the cytoplasmic/mitochondrial cyt-C significantly higher (*P* < 0.01) (Fig. 3c). These results confirmed that by upregulating the level of Bax/Bcl-2, the H_2_O_2_ could promote the mitochondria to release cyt-C to the cytoplasm and activate caspase-9 and caspase-3 which are the downstream apoptotic proteins of mitochondrial pathway. These results indicated that apoptosis stimulated by H_2_O_2_ at the early stage was mainly executed by endogenous apoptotic pathway (mitochondrial pathway) in the AF cells in vitro.

**Fig. 4:**
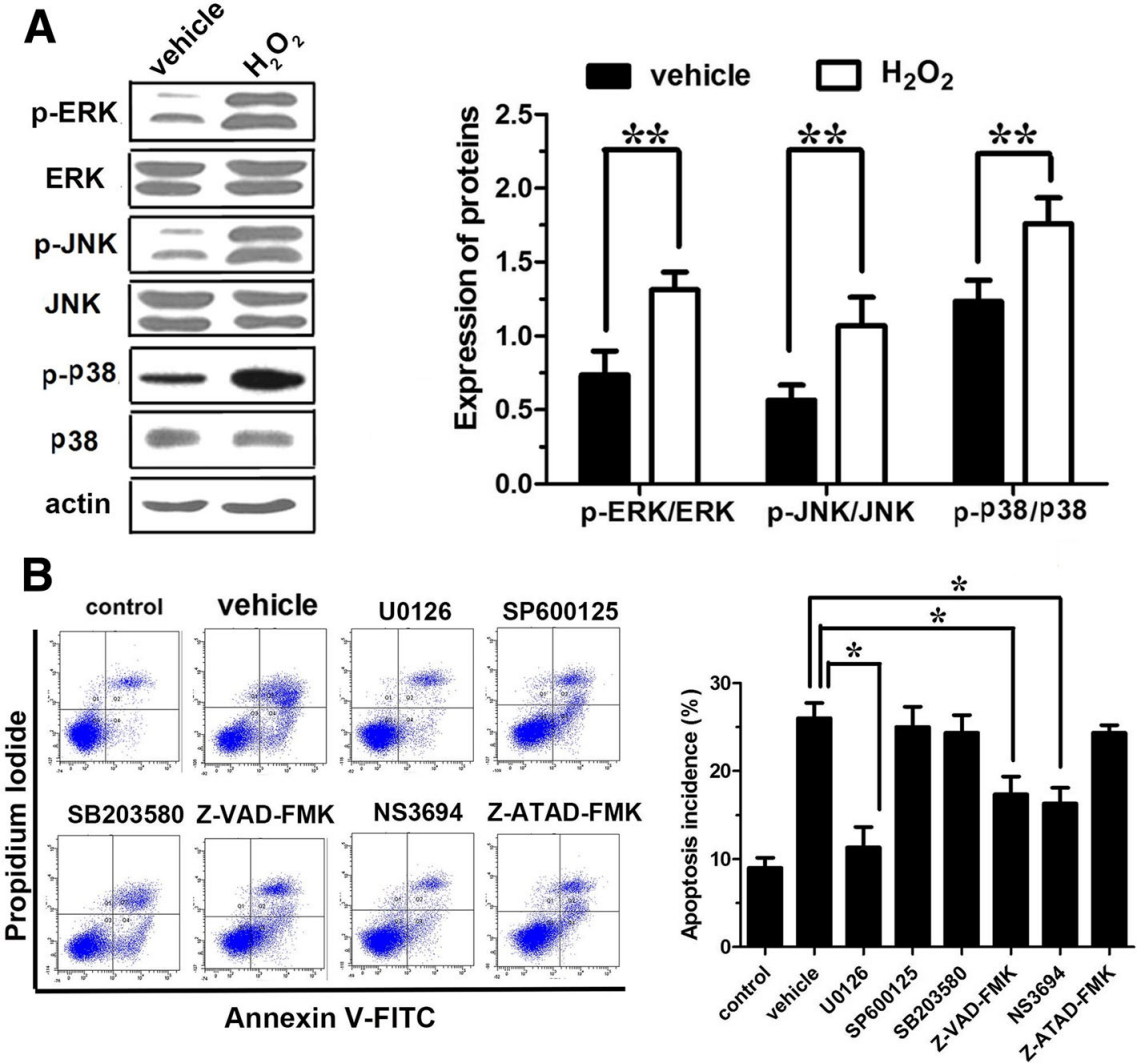
The pathways involved in H_2_O_2_-induced apoptosis in annulus fibrosus cells. **a** Western blot analysis to indicate the expressions of ERK1/2, JNK, and p38. **b** Flow cytometry analysis to investigate cell apoptosis after application of MAPK kinases blockers (ERK1/2: U0126, JNK: SP600125, p38: SB203580), caspase-3 blocker (Z-VAD-FMK), the mitochondrial pathway inhibitor (NS3694), and caspase-12 blocker (Z-ATAD-FMK). *P < 0.05; **P < 0.001

Oxidative stress also activates a variety of mitogen-activated protein kinases (MAPKs), including ERK1/2, JNK, and p38. Regulation of protein kinases is often located at upstream of autophagic and apoptotic signaling pathways. Thus, the expressions of ERK1/2, JNK, and p38 under 100 μmol/L H_2_O_2_ incubation for 1 h were further examined. Western blot analysis showed that the expressions of phosphorylated ERK1/2, JNK, and p38 were significantly increased (*P* < 0.01) (Fig. 4a). The results indicated that H_2_O_2_ might regulate upstream of autophagy and apoptosis by activating the MAPKs in the AF cells. To accurately verify our hypothesis, we further observed the apoptosis incidence by application of MAPK kinases blockers (ERK1/2: U0126, JNK: SP600125, p38: SB203580), caspase-3 blocker (Z-VAD-FMK), the mitochondrial pathway inhibitor (NS3694), and caspase-12 blocker (Z-ATAD-FMK). The Annexin V/PI double-staining experiments showed that U0126, Z-VAD-FMK, and NS3694 were able to significantly reduce H_2_O_2_-induced apoptosis in AF cells (*P* < 0.05) (Fig. 4b), while JNK, p38, and caspase-12 blockers had no effect on the apoptosis in AF cells (Fig. 4b). These results showed that activation of ERK1/2 may be the main mechanism for H_2_O_2_-induced apoptosis of AF cells through endogenous apoptotic pathway (mitochondrial pathway).

**Fig. 5:**
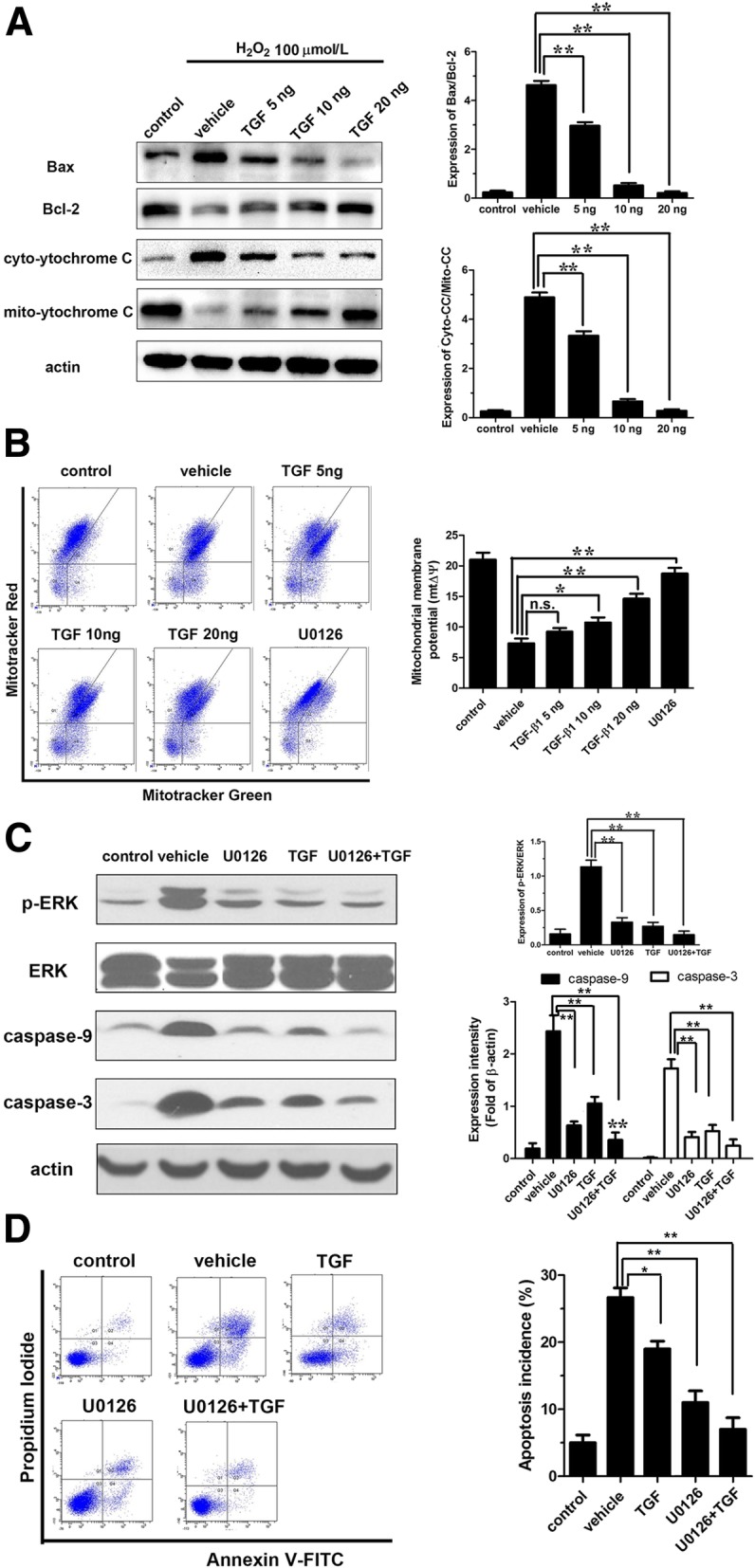
TGF-β1 inhibits the H_2_O_2_-induced apoptosis in annulus fibrosus cells. **a** Western blot to show the expression of Bax, Bcl-2, and cytoplasmic/mitochondrial cyt-C. **b** Mitochondrial membrane potential. **c** Western blot analysis to indicate the expressions of caspase-9 and caspase-3. **d** Flow cytometry analysis to investigate cell apoptosis. *TGF*-*β*1 transforming growth factor-β1, *H*_2_*O*_2_ hydrogen peroxide, *n*.*s* not significant; *P < 0.05; **P < 0.001

The effects of TGF-β1 (5, 10, 20 ng/mL) on mitochondrial apoptosis pathway were further determined. Western blot analysis showed that TGF-β1 could effectively reverse Bax/Bcl-2 level raised by H_2_O_2_ and exhibited a significantly statistical significance (*P* < 0.01) (Fig. 5a). Meanwhile, TGF-β1 could also significantly reduce the ratio of cytoplasmic/mitochondrial cyt-C raised by H_2_O_2_ (*P* < 0.01) (Fig. 5a). In addition, the results showed that 10 and 20 ng/mL of TGF-β1 could upregulate the mitochondrial membrane potential which decreased by H_2_O_2_ and had a significant dose-dependent manner (respectively, *P* < 0.05 and *P* < 0.01) (Fig. 5b), while ERK1/2 inhibitor U0126 could also significantly increase the mitochondrial membrane potential (*P* < 0.01) (Fig. 5b), indicating the potential association between TGF-β1 and ERK1/2. To further confirm our hypothesis, the expression of ERK1/2 was detected after TGF-β1 treatment alone or in combination with U0126. As anticipated, the expression of ERK1/2 was significantly decreased after TGF-β1 treatment, even slightly lower than U0126 (Fig. 5c). Meanwhile, the apoptosis rate and apoptosis-related proteins caspase-9 and caspase-3 were significantly declined (Fig. 5d). To sum up, these results demonstrated that the anti-apoptotic effects of TGF-β1 were mainly achieved through inhibiting the mitochondrial apoptosis via ERK1/2 signaling pathway.

### TGF-β1 protects against apoptosis through inhibition of H_2_O_2_-induced excessive autophagy in AF cells via ERK1/2 pathway

**Fig. 6:**
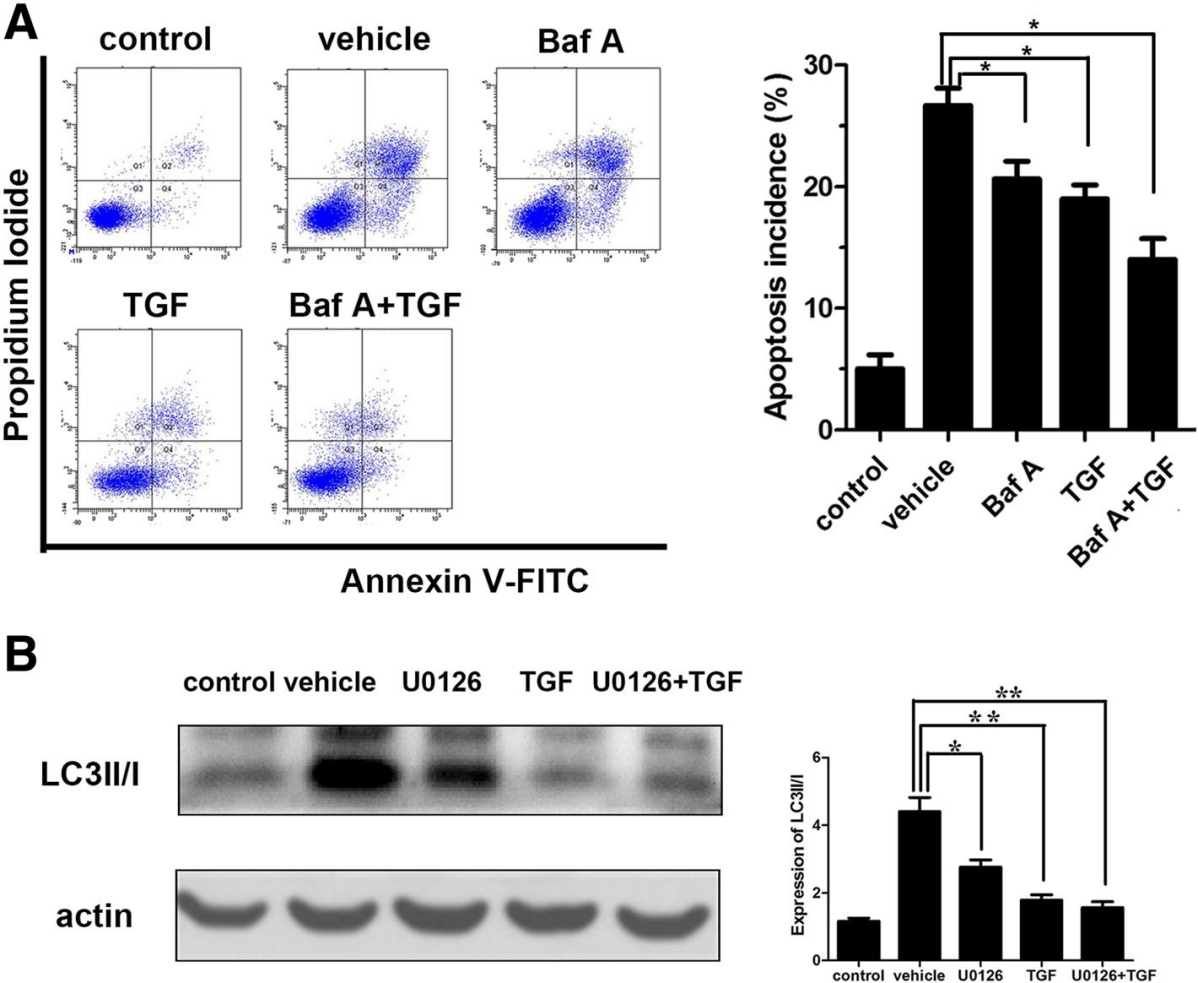
The relationship between autophagy and apoptosis. **a** Flow cytometry analysis to investigate cell apoptosis. **b** Western blot to show the expression of LC3 II/I. *TGF*-*β*1 transforming growth factor-β1, *Baf* Bafilomycin, autophagy inhibitor; U0126, ERK1/2 inhibitor; n.s, not significant; *P < 0.05; **P < 0.001

To further explore the relationship between autophagy and apoptosis, autophagy inhibitor Baf A (100 μM) or ERK1/2 inhibitor U0126 (10 μM) alone or in combination with TGF-β1 (20 ng/mL) were used to incubate the AF cells for 1 h. Flow cytometry showed that TGF-β1 and Baf A could partially reverse the apoptosis induced by H_2_O_2_ (*P* < 0.05). Moreover, the anti-apoptotic effect was more significant when combining them (*P* < 0.01) than either of them (*P* < 0.05) (Fig. 6a). These results confirmed that the excessive autophagy could increase apoptosis incidence in AF cells after stimulation with H_2_O_2_ for 1 h. Meanwhile, the inhibition effect of TGF-β1 on apoptosis was achieved partly through inhibiting autophagy. Furthermore, U0126 could reduce the expression of LC3 II/I (*P* < 0.01) (Fig. 6b). Thus, TGF-β1 may suppress H_2_O_2_-induced autophagy and then apoptosis through ERK1/2 pathway.

### TGF-β1 upregulates glutathione peroxidase and reduces the level of intracellular H_2_O_2_

**Fig. 7:**
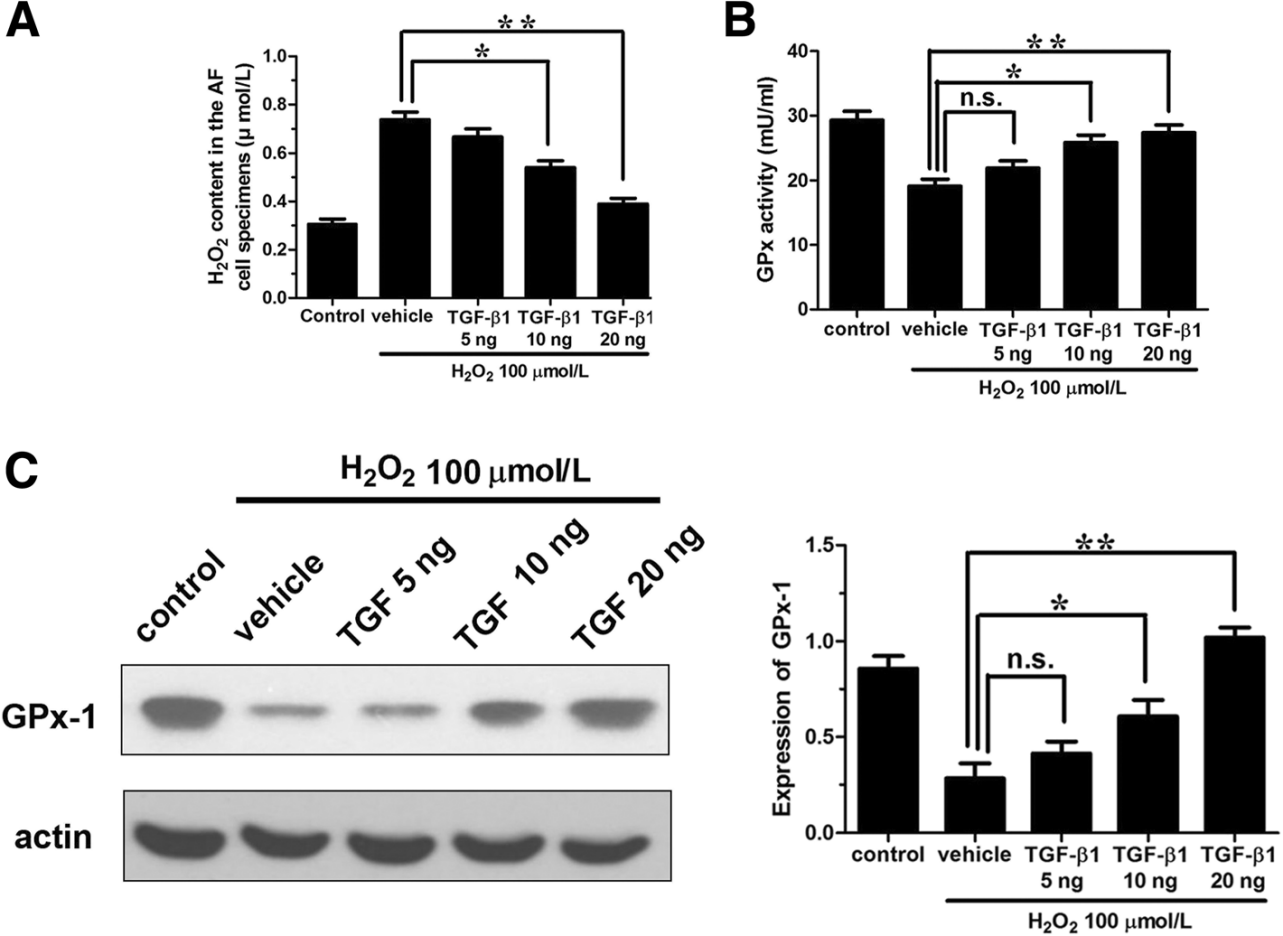
The antioxidants induction TGF-β1 in annulus fibrosus cells. **a** The level of intracellular H_2_O_2_. **b** GPx-1 activity. **c** The expression of GPx-1. *TGF*-*β*1 transforming growth factor-β1, *H*_2_*O*_2_ hydrogen peroxide, *GPx*-*1* glutathione peroxidase, *n*.*s* not significant; *P < 0.05; **P < 0.001

In addition to inhibit autophagy and then apoptosis, elimination of ROS and enhancement of antioxidants may be direct mechanisms for prevention of H_2_O_2_-induced cytotoxicity. Thus, the levels of intracellular H_2_O_2_ as well as GPx-1 and catalase activities were detected. It was found that, after exogenous H_2_O_2_ stimulation for 1 h, intracellular level of H_2_O_2_ was upregulated about 138% in AF cells than that in the control group (Fig. 7a). However, the addition of 10 and 20 ng/mL TGF-β1 could effectively reduce the level of intracellular H_2_O_2_ and had a dose-effect relationship (*P* < 0.05 and *P* < 0.01, respectively) (Fig. 7a). GPx-1 activity detection showed that H_2_O_2_ could decrease GPx-1 activity to 65% compared with the control group, but 10 and 20 ng/mL TGF-β1 could effectively increase the GPx-1 activity, which was also in a dose-effect manner (*P* < 0.05 and *P* < 0.01, respectively) (Fig. 7b). The promoting effect of TGF-β1 on GPx-1 expression was also further confirmed by Western blot analysis (Fig. 7c). However, the effect of TGF-β1 on catalase activity was not statistically significant and thus the data were not displayed.

## Discussion

A lot of studies have demonstrated that the ROS, which is generated from dysfunctional mitochondria of disc cells, is an important reason for activating autophagy and then apoptosis, promoting disc degeneration [21–24]. Hereby, inhibition of ROS-mediated biological processes may be important mechanisms for preventing IVDD. ROS can also be generated under starvation like amino acid deficiency and lack of energy to induce autophagy and apoptosis [25]. Therefore, to exclude the interference of malnutrition and lack of energy caused by starvation, only exogenous H_2_O_2_ was added to the AF cells to induce autophagy and apoptosis, and explore the protective roles of TGF-β1 on H_2_O_2_-treated cells. In line with our serum deprivation study [14], the present study also demonstrate TGF-β1 could partially reverse the toxicant effects of H_2_O_2_ on the viability of AF cells by inhibiting autophagy (showing reduced GFP-LC3 autophagosomes accompanied with decreased expressions of Beclin-1 and LC3 II/I and increased p62) and apoptosis (showing reduced expression of caspase-9 and caspase-3, Bax/Bcl-2, the ratio of cytoplasmic/mitochondrial cyt-C and MMP) at the early stage (0.5–4 h), preliminarily revealing that the supplementation of TGF-β1 may be an underlying treatment approach for IVDD [26].

Oxidative stress can activate the mitogen-activated protein kinases (MAPKs) pathway [27], including ERK1/2, JNK, and p38, to regulate cell autophagy and apoptosis. However, the main pathway may be different for different cells and the different drugs that induce or inhibit ROS-mediated autophagy and apoptosis. For example, Zhu et al. found Escin-activated ROS to upregulate p38 expression in a dose- and time-dependent manner and induced apoptosis and autophagy in human osteosarcoma cells, but had a minimal impact on JNK and ERK-2 [28]. Ki et al. observed chlorpyrifos-induced apoptosis by increasing ROS and activating JNK and p38 MAPK, but not ERK1/2 [29]. Lee et al. demonstrated p38, ERK, and JNK were all activated in ROS-related apoptosis by cudraflavone C [30]. Therefore, p38, ERK, and JNK were all detected in AF cells treated with H_2_O_2_ in our study. The results indicated that the expressions of phosphorylated ERK1/2, JNK, and p38 were significantly increased after H_2_O_2_ treatment. However, only the ERK1/2 inhibitor U0126, not JNK and p38 blockers reversed the H_2_O_2_ apoptosis in AF cells, indicating that activation of ERK1/2 may be the main mechanism for H_2_O_2_-induced apoptosis of AF cells. Also, the use of TGF-β1 can achieve the same effects as U0126 and there was a synergistic effect between TGF-β1 and U0126 to suppress the ERK1/2, apoptosis, and autophagy, further illustrating that TGF-β1 may inhibit H_2_O_2_-induced apoptosis and autophagy by the ERK1/2 pathway. This seemed to be in line with previous studies in renal epithelial cells where apoptosis decreased if ERK was blocked [31, 32], but contrary with our previous research that TGF-β1 could enhance the expression level of p-ERK to inhibit autophagy and apoptosis in AF cells under serum deprivation in vitro [14]. This may be attributed to the dual roles of ERK1/2 [33]. Moreover, apoptosis and autophagy are two main programmed cell death patterns and closely linked [34]. Some studies suggested that autophagy could inhibit or delay the occurrence of apoptosis [35, 36], but the other indicate that autophagy may promote apoptosis [37, 38]. Thus, investigation of the relationship between autophagy and apoptosis in AF cells after H_2_O_2_ or TGF-β1 treatment is indispensable. In this study, we found that the apoptosis incidence was significantly declined after the use of autophagy inhibitor Baf A and the effect was more obvious when the combination of Baf A and TGF-β1, implying that the increased autophagy may promote apoptosis and TGF-β1 may inhibit apoptosis by blocking autophagy. This finding seemed to be in accordance with our previous study in nucleus pulposus cells [12]. Accordingly, we believe that TGF-β-ERK-autophagy-apoptosis pathway may be an important mechanism to prevent oxidative stress-induced AF cell loss and the development of IVDD.

The previous studies reported that ROS generated within cells can be neutralized by superoxide dismutase, glutathione peroxidase, catalase or other non-enzymatic antioxidants and glutathione, etc., and achieved the balance of oxidation-reduction in cells [39, 40]. Thus, the increased apoptosis after H_2_O_2_ treatment may be attributed to the lower generation of antioxidants and TGF-β may alleviate ROS-induced injuries in AF cells by increasing the formation of antioxidants. In this study, the activity of GPx-1 was also detected. As expected, the GPx-1 activity and expression were significantly enhanced after the addition of medium-high dosed TGF-β1, which was also coincident with the previous studies in colon cancer cells [41]. The regulation mechanisms of TGF-β on the expression and activity of antioxidant enzymes (such as GPx-1) may be also attributed to the activation of Nrf2 (nuclear factor erythroid 2 [NF-E2]-related factor 2) transcription pathway [42, 43], although it should be further confirmed in our subsequent study.

## Conclusion

Our findings suggested that exogenous H_2_O_2_ had significant cytotoxicity, and promoted autophagy and apoptosis through ERK pathway in rat AF cells. TGF-β1 could reduce the toxic effects of H_2_O_2_ to the AF cells through inhibiting ERK pathway to inhibit excessive autophagy and then apoptosis induced by H_2_O_2_. The protective effects of TGF-β1 on rat AF cells may also be achieved by upregulating GPx-1 and reducing the level of intracellular H_2_O_2_. These findings indicated the supplementation of TGF-β1 (or drugs that induced the increase of TGF-β1) may be an underlying approach for prevention and alleviation of AF cell loss and subsequent development and progression of IVDD and LBP in clinic.

## Abbreviations

AF: Annulus fibrosus
GPx-1: Glutathione peroxidase
IVDD: Intervertebral disc degeneration
LBP: Lower back pain
MAPKs: Mitogen-activated protein kinases
MMP: Mitochondrial membrane potential
NO: Nitric oxide
ROS: Reactive oxygen species
TGF-β1: Transforming growth factor-β1
